# MONKEY: identifying conserved transcription-factor binding sites in multiple alignments using a binding site-specific evolutionary model

**DOI:** 10.1186/gb-2004-5-12-r98

**Published:** 2004-11-30

**Authors:** Alan M Moses, Derek Y Chiang, Daniel A Pollard, Venky N Iyer, Michael B Eisen

**Affiliations:** 1 Graduate Group in Biophysics, University of California, Berkeley, CA 94720, USA; 2 Center for Integrative Genomics, University of California, Berkeley, CA 94720; 3 Department of Molecular and Cell Biology, University of California, Berkeley, CA 94720, USA; 4 Department of Genome Sciences, Genomics Division, Ernest Orlando Lawrence Berkeley National Lab, 1 Cyclotron Road, CA 942770, USA

## Abstract

We introduce a method (MONKEY) to identify conserved transcription-factor binding sites in multispecies alignments. MONKEY employs probabilistic models of factor specificity and binding-site evolution, on which basis we compute the likelihood that putative sites are conserved and assign statistical significance to each hit. Using genomes from the genus *Saccharomyces*, we illustrate how the significance of real sites increases with evolutionary distance and explore the relationship between conservation and function.

## Abstract 2

MONKEY is a new method for identifying conserved transcription-factor binding sites from multiple-sequence alignments.

## Background

Different types of genomic features have characteristic patterns of evolution that, when sequences from closely related organisms are available, can be exploited to annotate genomes [1]. Methods for comparative sequence analysis that exploit variation in rates and patterns of nucleotide evolution can identify coding exons [1,2], noncoding sequences involved in the regulation of transcription [3,4] and various types of RNAs [5-7]. While most of these methods have been developed for and applied to pairwise comparisons, sequence data are increasingly available for multiple closely related species [8]. It is therefore of considerable importance to develop sequence-analysis methods that optimally exploit evolutionary information, and to explore the dependence of these methods on the evolutionary relationships of the species in comparison.

Sequence-specific DNA-binding proteins involved in transcriptional regulation (transcription factors) play a central role in many biological processes. Despite extensive biochemical and molecular analysis, it remains exceedingly difficult to predict where on the genome a given factor will bind. Transcription factors bind to degenerate families of short (6-20 base-pairs (bp)) sequences that occur frequently in the genome, yet only a small fraction of these sequences are actually *bona fide *targets of the transcription factor [9]. A major challenge in understanding the regulation of transcription is to be able to distinguish real transcription factor binding sites (TFBSs) from sequences that simply match a factor's binding specificity. Because the evolutionary properties of TFBSs are expected to be different from their nonfunctional counterparts, comparative analyses hold great promise in helping to address this challenge.

In the past few years, several methods have been introduced to identify conserved (and presumably functional) TFBSs for a factor of known specificity (in contrast to the larger set of methods that use comparative data in motif discovery or to otherwise identify sequences likely to be involved in *cis*-regulation). Each of these methods explicitly or implicitly adopts one of several distinct definition of a conserved TFBS. These include a binding site in a reference genome that is perfectly or highly conserved [8,10-12]; a binding site in a reference genome that lies in a highly conserved region [4]; or a position at which the binding model predicts a binding site in all species [13-18].

In a previous study we characterized the evolution of experimentally validated TFBSs in the *Saccharomyces cerevisiae *genome, finding that functional TFBSs evolve more slowly than flanking intergenic regions, and more strikingly, that there is considerable position-specific variation in evolutionary rates within TFBSs [19]. We further showed that evolutionary rate at each position is a function of the selectivity of the factor for bases at that position.

Our goal here is to incorporate these specific evolutionary properties of TFBSs into the search for conserved TFBSs. Or, more precisely, to develop a method that, given the specificity of a transcription factor, identifies conserved binding sites in multiple alignments by taking into account the sequence specificity and patterns of evolution expected for TFBSs, while still fully exploiting the phylogenetic relationships of the species being compared.

In addition to developing new methods, there are several hypotheses regarding the comparative annotation of TFBSs that we are interested in testing. It has been noted that the effectiveness of such analyses will depend critically on the evolutionary distance separating the species used. At very close distances TFBSs will appear conserved because there has been insufficient time for substitutions to occur. As distance increases, and substitutions occur most rapidly at nonfunctional positions, our ability to detect constrained binding sites should improve until we are no longer able to reliably assign orthology based on sequence alignment. To overcome this problem of divergence distances exceeding what can be aligned, the sequences of multiple closely related species can be used to span the same evolutionary distances (and presumably provide the same discriminatory power) as fewer more distantly related ones. However, aside from these qualitative expectations, the dependence of the ability to identify conserved TFBSs on evolutionary distance and tree topology has not been rigorously investigated. Because the software MONKEY can be applied to multiple alignments of varying numbers of species and produces scores that can be meaningfully compared across different sets of species, we are now able to address these issues.

## Results

### Overview

We developed an approach to identify conserved TFBSs that combines probabilistic models of binding-site specificity [20-22] with probabilistic models of evolution [23,24]. Starting with an alignment of sequences from multiple related species, we use the known sequence specificity for a transcription factor to compare the likelihood of the sequences under two evolutionary models - one for background and one for TFBSs. The central feature of this method that underlies its ability to identify conserved TFBSs is that it uses a specific probabilistic evolutionary model for the binding sites of each transcription factor. The evolutionary model we use for TFBSs [25] assumes that sites were under selection to remain binding sites throughout the evolutionary history of the species being studied. This model uses the sequence specificity of the factor to predict patterns and rates of evolution that recapitulate the patterns and rates observed in real TFBSs [19].

### MONKEY: scanning alignments to identify conserved transcription factor binding sites

MONKEY, our tool for identifying conserved TFBSs, takes as input a multiple sequence alignment, a tree describing the relationship of the aligned species, a model of a transcription factor's binding specificity and a model for background noncoding DNA. It returns, for each position in the alignment, a likelihood ratio comparing the probability that the position is a conserved binding site for the selected factor compared to the probability that the position is background.

### Extending matrix searches to multiple sequence alignments

For the model of binding specificity, we use a traditional frequency matrix [20-22]. The values in the matrix - *f*_*ib *_- represent the probability of observing the base *b *(A, C, G or T) at the *i*th position in a binding site of width *w*. For the model of the background, we use a single set of base frequencies *g*_*b*_.

A widely used statistic for scoring the similarity of a single sequence to a frequency matrix is the log likelihood ratio comparing the probability of having observed a sequence *X *of width *w *under the motif model (a frequency matrix, designated as *motif*) to the probability of having observed *X *under the background model (designated by *bg*), which can be easily reduced to:



where *X*_*ib *_is an indicator variable which equals 1 if base *b *is observed at position *i*, and zero otherwise.

This classifier can be motivated by the approximation that the data are distributed as a two-component mixture of sequences matching the frequency matrix and sequences drawn from a uniform background. In practice, we compute this score using a position-specific scoring matrix (PSSM) with entries, *M*_*ib *_= log(*f*_*ib*_/*g*_*b*_), and find *S *for a particular *w*-mer by adding up the entries that correspond to the bases in the query sequence.

In extending this to a pair of aligned sequences *X *and *Y*, we want to perform the same calculation on their common ancestor *A*. Since *A *is not observed, we consider all possible ancestral sequences by summing over them, weighting each by their probability given the data (*X *and *Y*), the phylogenetic tree (*T*) that relates the sequences, and a probabilistic evolutionary model [23].

We can write a new score representing the log-likelihood ratio that compares the hypothesis that *X *and *Y *are a conserved example of the binding site represented by the frequency matrix to the hypothesis that they have been drawn from the background:



where *R*_*motif *_and *R*_*bg *_are rate matrices describing the substitution process of the binding site and background respectively. Using the conditional independence of the sequences *X *and *Y *on the ancestor, *A*, and writing *T*_*AX *_for the evolutionary distance separating sequence *X *from *A*, this becomes:



The class of evolutionary models used by MONKEY define a substitution matrix, *p*(*X*_*i*_|*A*_*i*_, *t*) = *e*^*Rt*^, that represents the probability of observing each base at position *i *in the extant sequence (*X*) given each base in the ancestral sequence (*A*) after *t *units of evolutionary time or distance, given some rate matrix, *R *[23]. Since these models retain positional independence, we can rewrite this as:



This can be extended to more than two sequences, that is, (*X*, *Y*, ..., *Z*), by replacing the probabilities of *X *and *Y *with the probability with the left and right branches of the tree below, and performing the calculation at the root. The probabilities of the left and right branches of the tree can be calculated recursively as has been described previously [23].

Once again, for practical purposes we can convert these scores to a PSSM, whose entries are given for the pairwise case by:



where at each position we now index by the bases *a *and *b *in the two sequences. For multiple alignments of *n *species, each position requires 4^*n *^entries.

### Evolutionary models

The use of evolutionary models is critical to the function of MONKEY. Myriad of such models exist, and in principle all can be used in MONKEY. For the background, it is natural to use a model appropriate for sites with no particular constraint, such as the average intergenic or synonymous rates. MONKEY allows the use of the JC [26] or HKY [27] models, and here we use the latter with the base frequencies, rates and transition-transversion rate-ratio estimated from noncoding alignments assuming a single model of evolution over the noncoding regions (see details in Materials and methods). It is also possible to estimate the evolutionary model separately for each intergenic alignment, although the small size of yeast intergenic regions leads to variable estimates.

In principle, the JC and HKY models can also be used for the motif, with rates set according to our expectation of the overall rate of evolution in functional binding sites, which has been estimated as two to three times slower than the average intergenic rate [19]. However, we have previously shown that there is position-specific variation in evolutionary rates within functional transcription factor binding sites [19] and that positions in a motif with low degeneracy in the binding-site model evolve more slowly than positions with high degeneracy; this relationship between the equilibrium frequencies and the position-specific evolutionary rates is accurately predicted by an evolutionary model from Halpern and Bruno (HB model) [25].

In using this model, we assume that sequences evolve under constant purifying selection to maintain a particular set of equilibrium base frequencies. The use of this model corresponds to a definition of a conserved TFBS as a sequence position where there has always been a binding site for the transcription factor. Although the model does not strictly require that a binding site be present in each of the observed species, positions lacking such sites will have lower probabilities as they require the use of less probable substitutions. The rate of change from residue a to b at position *i *in the motif is given by:



where *Q *is the (position independent) underlying mutation matrix, which we set equal to the background model (*Q *= *R*_*bg*_), and *f *is the frequency matrix describing the specificity of the factor. Thus, for each position in the motif, the HB model predicts the rates of each type of substitution as a function of the frequency matrix, and the background model.

### Comparing hits for different factors and evolutionary distances: computing the null distribution

To compare scores from different evolutionary distances and different factors, it is critical that we are able to assign significance to a particular value of the score. To do so, we need to compute the distribution of the score under the null hypothesis that the sequence is part of the background. Calculating a *p*-value for a score *S *in a single sequence requires the enumeration of all possible *w*-mers that have a score *S *or greater under the background model. For *n *aligned sequences this requires the enumeration all 4^*wn *^possible sets of aligned *w*-mers with scores *S *or greater under the background model. While the number of possible alignments of *n w*-mers can be unmanageably large for even small values of *n *and *w*, because we treat each position independently we can enumerate these possibilities efficiently using an algorithm developed for matrix searches of single sequences [28,29].

Every observed score is a sum of *w *numbers, one from each column of the matrix. The probability of observing exactly score *S *is the number of paths through the matrix whose entries add up to *S*, weighted by the probability of the path. By converting the matrix to integers, we can compute this probability for all values of *S *recursively. We initialize *P*_*i*_(*S*) (the probability of observing score *S *after *i *columns in the matrix) by setting *P*_*0*_(*S*) = 1 for *S *= 0, and *P*_*0*_(*S*) = 0 for *S *≠ 0. We then compute the values of the function for *i *= [1, *w*] as follows:



For aligned sequences, *c *represents a column in the alignment, and the sum is over all 4^*n *^possible columns an alignment of *n *sequences. The probability distribution function (PDF) of scores is *P*_*w*_(*S*), and from this the cumulative distribution function (CDF), the probability of observing a score of *S *or greater, can be directly computed. Although in principle we can compute the probabilities to arbitrary precision, because the time complexity increases with the number of possible scores, we limit the precision to within approximately 0.01 bits.

**Figure 1:**
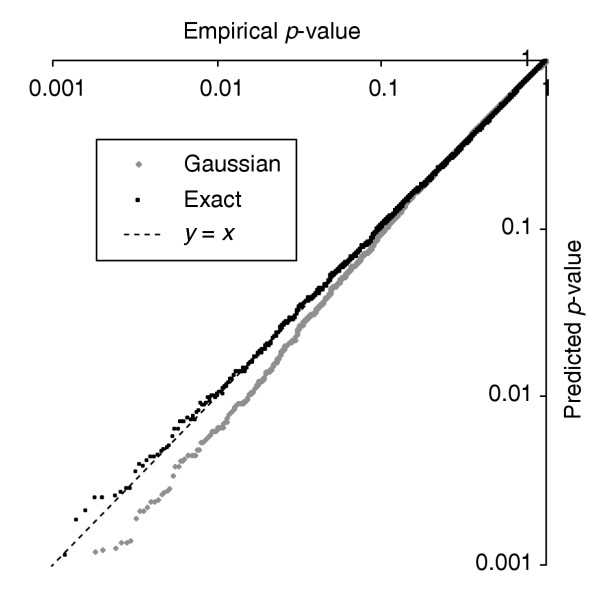
Accuracy of *p*-value estimations. To examine the accuracy of our *p*-value estimates, we compared the empirical *p*-value (computed from the observed distribution of scores) to *p*-values computed using either the exact method described above (black points) or Gaussian approximation (gray points). The scores represent the simple score at a distance of 0.1 substitutions per site calculated using the Gcn4p matrix from SCPD [33]. Other models and matrices produce similar results.

Figure 1 compares empirical *p*-values from 5,000 pairs of sequences evolved in a simulation (see Materials and methods) with those computed by this method, and shows that they agree closely. We have used this method to compute the CDFs for alignments of up to six species, and therefore can apply our method to most comparative genomics applications. We note, in addition, that the likelihood ratio scores are approximately Gaussian (data not shown). As the means and variance of the scores under each model can be computed efficiently (see Materials and methods) we can estimate *p*-values using a Gaussian approximation (Figure 1) when the number of sequences in the alignment is large.

### Heuristics for alignments with gaps

The treatment of alignment gaps in identifying conserved TFBSs is somewhat problematic. One the one hand, nonfunctional sequences may be inserted and deleted over evolution more rapidly than functional elements [30-32], and thus the presence of a gap aligned to a predicted binding site could indicate that it is nonfunctional. On the other hand, alignment algorithms are imperfect, and must often make arbitrary decisions about the placement of gaps. We sought to design a heuristic that accommodated both these aspects of genomic sequence data by locally optimizing alignments for the purpose of comparative annotation of regulatory elements.

The idea is to assign a poor score to regions of the alignment with a large number of gaps, but to locally realign regions with a small number of gaps to identify conserved but misaligned binding sites. To do this, we scan along the ungapped version of one of the aligned sequences - the 'reference' sequence. For each position in the reference sequence *p*_*r*_, we define a window in each other sequence around *p*_*s*_, the position in sequence *s *aligned to position *p*_*r*_. The window runs from *p*_*s *_- (*a *+ *b*) to *p*_*s *_+ *w *+ (*a *+ *b*), where *a *and *b *are the number of gaps in the aligned versions of sequences *r *and s in position *p *to *p *+ *w*, where *p *is the position in the alignment of *p*_*r*_. For each subsequence of length *w *in the window, we calculate the percent identity to the reference sequence, and create an alignment of *p*_*r *_to *p*_*r *_+ *w *(in the reference sequence) to the most similar word in the window of each other sequence. This locally optimized alignment is then scored. Note that if *a *and *b *are zero (meaning there are no gaps in the aligned sequence), no optimization is done. If *a *is too large (in most contexts greater than five) we exclude that region of the alignment from further. This heuristic encapsulates the idea that too many gaps are indicative of lack of constraint, but conservatively allows for a few gaps due to alignment or sequence imperfections.

### Application to *Saccharomyces*

The genome sequences of several species closely related to the budding yeast *Saccharomyces cerevisiae *have recently been published and become models for the comparative identification of transcription factor binding sites [8,11]. We aligned the intergenic regions of *S. cerevisiae *genes to their orthologs in *S. paradoxus*, *S. mikatae*, *S. bayanus *and *S. kudriavzevii *genomes using CLUSTALW (see Materials and methods) and sought to evaluate the effectiveness of MONKEY under different evolutionary models and distances.

Ideally, we would use several diverse transcription factors with known binding specificity, where the set of matches to the factor's matrix in the *S. cerevisiae *genome could be divided into two reasonably sized sets: those known to be bound by the factor (positives) and those known not to be bound by the factor (negatives). Unfortunately, even in yeast, the number of such cases is limited. For many factors we can identify true positives by combining high- and low-throughput experimental data that supports the hypothesis that a particular position in the genome is bound by a given factor. A true negative set, however, must be constructed on the basis of lack of evidence that a sequence is functional, as the interpretation of negative results almost always is ambiguous. In the case of transcription factor binding sites this is particularly problematic, because DNA-binding proteins have overlapping specificity, and we may therefore observe conservation of a binding site because it is bound by another factor with similar specificity. After evaluating all factors with binding specificity in *Saccharomyces cerevisiae *Promoter Database (SCPD) [33], we focus on Gal4p and Rpn4p for further analysis (see Table 1 for properties of these factors, and Materials and methods for a description of the selection of positive and negative sets).

### The effects of evolutionary models on the discrimination of functional binding sites

To evaluate the performance of our evolutionary method in correctly identifying *bona fide *binding sites, we calculated the *p*-values of the positive and negative sites for each factor, using MONKEY on alignments of all five genomes for Rpn4p and four species (with *S. kudriavzevii *excluded because too few sequences were available) for Gal4p. We compared the performance of MONKEY with the HB model to scores from *S. cerevisiae *alone and to a 'simple' score (equal to the average of the single sequence log likelihood ratios) that utilizes all the comparative data without an evolutionary model.

The results are summarized in Table 2. An ideal scoring method would assign low *p*-values to real sites (positives) and high *p*-values to spurious sites (negatives), and we therefore compared the *p*-values assigned by monkey based on the HB model to those based on the 'simple' score. Not surprisingly, both methods were a great improvement over searching in *S. cerevisiae *alone. Overall, when compared to each other, the HB score assigned lower *p*-values to the binding sites more often in the positive sets (90% for Gal4p and 80% for Rpn4p) and less often in the negative sets (20% for Gal4p and 25% for Rpn4p) than did the simple score. We note that some of the supposedly functional Rpn4p sites were assigned higher *p*-values in *S. cerevisiae *alone, suggesting that they are not in fact conserved; these will be discussed below.

### The effect of evolutionary distance on the discrimination of functional binding sites

**Figure 2:**
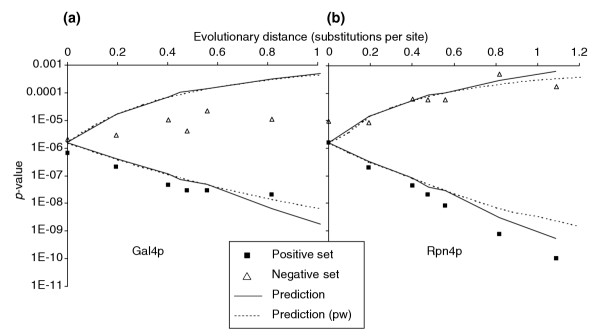
Significance of matches increases with evolutionary distance. Median *p*-values for the positive (black squares) and negative (white triangles or white triangle points) sets of binding sites for **(a) **Gal4p and **(b) **Rpn4p at different evolutionary distances represented by comparing *S. cerevisiae *to different subsets of the available species. For both factors, as evolutionary distance increases, the median *p*-value of the functional matches decreases, indicating that they are less likely to have appeared by chance. Conversely, the median *p*-value of the nonfunctional matches (negative set, white symbols) increases. These observations agree with our predictions for the behavior of the *p*-values (solid traces) under either the HB evolution for the motif or HKY evolution for the background. There is little difference between these predictions and similar ones that assume that all the comparisons were pairwise (dotted traces).

As evolutionary distance increases, we expect fewer matches to the matrix to be conserved by chance, which implies that the probability of observing matches as highly conserved as the functional sites should decrease. Similarly, we expect the nonfunctional sites to show many substitutions and their *p*-values to increase over evolution. To explore the change in *p*-values over evolutionary distance, we scored the functional and nonfunctional sets of binding sites at a variety of evolutionary distances by creating alignments of different combinations of species (see Materials and methods). The median *p*-value of the positive set of TFBSs decreases monotonically with evolutionary distance, with the rate of decrease an approximately constant function of evolutionary distance (see Figure 2). The median *p*-value for the binding sites in the negative set increases with evolutionary distance, although somewhat erratically. This demonstrates that MONKEY effectively exploits evolutionary distance, and confirms our intuition that as evolutionary distance increases, functional elements should be increasingly easy to distinguish from spurious predictions.

To test this hypothesis on a more quantitative level we sought to compare the observed scores with the expected scores assuming that binding sites evolved precisely according to the evolutionary models used by MONKEY. Briefly, given a binding-site model and a phylogenetic tree, we assume we have observed a binding site in the reference genome, and that this site evolves along the tree under either the motif model (HB) or background model (HKY), representing functional and nonfunctional binding sites, respectively (see Materials and methods for details). The expected *p*-values associated with the functional binding sites (Figure 2, solid lines) showed reasonable agreement with the models, consistent with previous observations that they are evolving under constraint that is well modeled by the purifying selection on the base frequencies in the specificity matrix [19].

### Pairwise versus multi-species comparisons

The comparisons at the different evolutionary distances used in Figure 2 employed variable numbers of species, with the shorter distances representing primarily pairwise comparisons and the longer distances comparisons of three or more species. While we expect the variation in *p*-values with different combinations of species to be primarily a function of the evolutionary distance spanned by these species, there will also be effects related to the number of species and the topology of the three. For example, in the limit of very long branch lengths, the evolutionary *p*-values are on the order of the power of the number of species and are independent of evolutionary distance. In contrast, in the limit of very short branch lengths, the evolutionary *p*-values depend only on the distance spanned by the comparison, as most of the information provided by additional species is redundant. However, because most comparisons that are actually carried out are far from either of these extremes, we sought to evaluate the effects of species numbers and tree topology for the *Saccharomyces *species analyzed here.

First, we recomputed the expected *p*-values for all the distances analyzed in Figure 2, except that instead of using the real tree topology, we used a single pairwise comparison at the same evolutionary distance (Figure 2, dotted lines). For example, for the Rpn4p analyses using all five species we assumed a pairwise comparison at an evolutionary distance of around 1.1 substitutions per site. Note that this is considerably more distant than any of the pairwise comparisons available among these species. The predictions for the pairwise and multi-species comparisons are very similar, suggesting that at the evolutionary distances spanned by these species there is little difference in using multiple species alignments relative to a pairwise alignment that spans the same evolutionary distance. Only at the longest distances considered (greater than 0.8 substitutions per site) does the power of the pairwise comparison begin to level off, although there are other reasons that multiple species comparisons might still be preferred (see Discussion).

**Figure 3:**
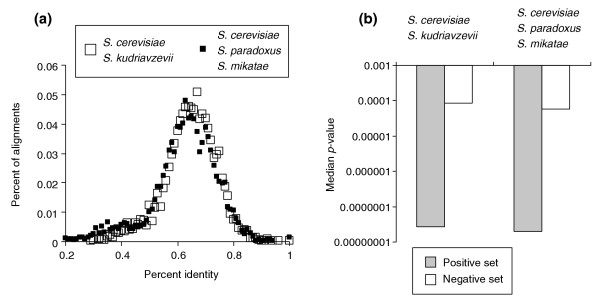
Significance of binding sites in pairwise or three-way comparisons at similar evolutionary distance. **(a) **Histogram of the percent identities of all aligned noncoding regions of *S. cerevisiae *and *S. kudriavzevii *(open squares) and *S. cerevisiae*, *S. paradoxus *and *S. mikatae *(filled squares). **(b) **Median *p*-values of functional matches (positive set, gray bars) and the nonfunctional matches (negative set, open bars) for *S. cerevisiae *and *S. kudriavzevii *alignments (left) and *S. cerevisiae*, *S. paradoxus *and *S. mikatae *alignments (right). The similarity of these *p*-values supports the idea that multiple similar genomes can be used to span longer evolutionary distances, but at these close evolutionary distances provide little additional power.

To complement this theoretical analysis, we were interested in using empirical data to compare pairwise and multi-species analyses. Fortuitously, the evolutionary distance between *S. cerevisiae *and *S. kudriavzevii *is almost exactly equal to the evolutionary distance spanned by *S. cerevisiae*, *S. paradoxus *and *S. mikatae *(median tree length approximately 0.5 substitutions per site; see Figure 3a). Because our models predict that we are in a regime where evolutionary distance is the primary determinant of the *p*-values, we expect searches using these different sets of species to yield similar results. We tested this hypothesis by calculating the *p*-values associated with the Rpn4p-binding sites using the sequences from these two comparisons. The median *p*-values in both the positive and negative sets are very similar (Figure 3b), confirming that at these relatively short evolutionary distances, the power of the comparative method is independent of the number of species considered (see Discussion).

Taken together, these results strongly support the idea that when appropriate methods are used, data from multiple species can be combined effectively to span larger evolutionary distances. Note that this in no way implies that the addition of extra species to an existing pairwise comparisons is not useful - such additions will always increase the evolutionary distance spanned by the species and thus will increase the power of the comparison.

### Testing the power of comparative annotation of transcription factor binding sites

At the distances spanned by all available sequence data, the *p*-values are so small that we no longer expect to find matches of the quality of those in the positive set by chance, especially for Rpn4p. To test this further, we scanned both strands of all the available alignments of all five *sensu stricto *species (around 2.7 Mb) to identify our most confident predictions of conserved matches to the Rpn4p matrix. We chose the *p*-value cutoff of 1.85 × 10^-8^, which corresponds to a probability of 0.05 of observing one match at that level over the entire search (using a Bonferroni correction for multiple testing). After excluding divergently transcribed genes, there were 56 genes that contained putative binding sites at that *p*-value. Of 32 genes in our positive set that had sequence available for all five species, 30 had binding sites below this *p*-value. Of the 28 genes in the negative set for which sequences were available, only three had binding sites below this cutoff. In this (nearly ideal) case we have ruled out nearly 90% of the negative set at the expense of less than 10% of the positives.

**Figure 4:**
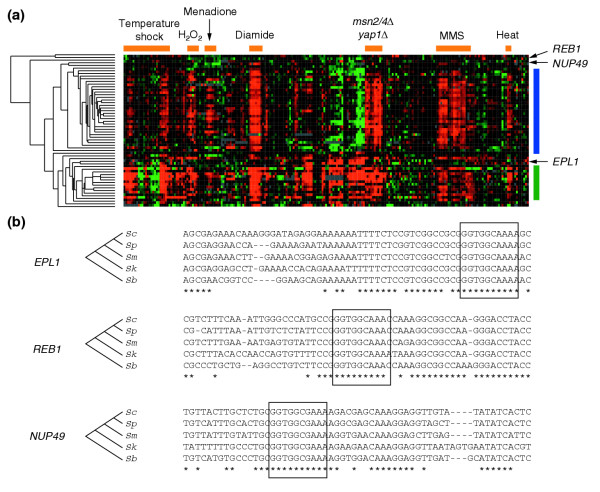
Relationship between conserved Rpn4p-binding sites and expression. **(a) **We identified 56 Rpn4p-binding sites with *p*-values below 1.85 × 10^-8 ^using all five species and the HB model. The expression patterns of these genes (clustered and displayed as in [44]) fall into two major groups: the 'stereotypical' proteasomal pattern (indicated by a blue bar at the right), and a second group expressed in these and additional conditions (indicated by the green bar). The orange bars above the expression data correspond to (left to right) temperature changes, treatment with H_2_O_2_, treatment with the superoxide generating drug menadione, treatment with the sulfhydryl oxidant diamide, deletions of *YAP1 *and *MSN2/4*, treatment with the DNA damaging agent methylmethanesulfonate (MMS), and heat shock in deletions of *MEC1 *and *DUN1 * [48,49]. **(b) **Examples of conserved Rpn4p sites (boxed) that do not fall in either expression group (neither blue nor green bar).

Examining the expression patterns of these genes (Figure 4a) allows them to be divided into three major classes. The first is a group (indicated by a blue bar) containing 30 genes (28 of which were in our original positive set and two other genes) that show a very similar pattern over the entire set of conditions. The second group (indicated by a green bar) contains 11 genes (of which only one was in our original positive set) that show uncoordinated gene expression changes in some conditions in addition to the stereotypical Rpn4p expression pattern. It is possible that these genes' regulation is controlled by multiple mechanisms under different conditions [34], and regulation by Rpn4p is one contribution to their overall pattern of expression. Further supporting this hypothesis, only one of these genes (*UFD1*) is annotated as involved in protein degradation, and three (*YBR062C*, *YOR052C *and *YER163C*) have unknown functions.

Finally, and most surprising from the perspective of comparative annotation, is a third set of 14 genes, including one from our original positive set and three from our negative set, most of which show no evidence of the proteasomal expression pattern associated with Rpn4p (Figure 4b). It is extremely unlikely that these sequences have been conserved by chance, and we suggest that they represent matches that are conserved for reasons other than binding by Rpn4p (see Discussion).

### Nonconserved binding sites in regulated genes

**Figure 5:**
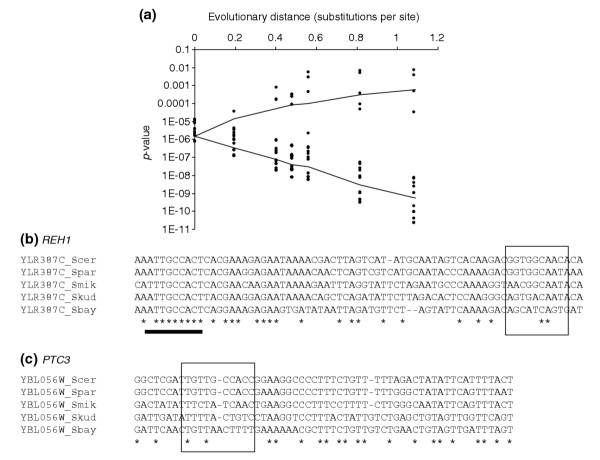
Some apparently functional Rpn4p-binding sites are not conserved. **(a) **The MONKEY *p*-values (points) of all putatively functional Rpn4p-binding sites at varying evolutionary distances, along with the expected values under the HB and HKY models (solid traces). The majority of sites behave as expected for conserved binding sites (lower trace). Several, however, behave as expected for unconstrained sites (upper trace). **(b) **The predicted binding site (indicated by a box) in *REH1*, which encodes a protein of unknown function in *S. cerevisiae*, is not conserved, whereas a binding site with a lower score is conserved (indicated by a black bar). **(c) **A very poorly conserved match upstream of *PTC3*; in this case no other sites can be found in the region.

Having identified examples of conserved binding sites whose nearby genes showed no evidence of function, we decided to examine the converse: binding sites near regulated genes, and therefore presumably functional, that are not conserved. Figure 5 shows the *p*-values of individual positive Rpn4p sites at different evolutionary distances. While most of the sites follow the trajectory predicted for sites evolving under the HB model, the *p*-values for four of the positive sites seem to be well-modeled by the 'background' or unconstrained model. This is surprising because we expect these binding sites to be functional, and therefore under purifying selection. One explanation is that some of these sites may have been misannotated as functional. For example, in addition to a nonconserved positive site, the upstream region of *REH1 *contains another binding site that is a weaker match to the Rpn4p matrix (Figure 5b) and did not pass our threshold for inclusion in the positive set (see Materials and methods). This weaker match is more highly conserved and may represent the functional site in this promoter. In the case of *PTC3*, however, we can find no other candidate binding sites nearby (Figure 5c). This represents a possible example of binding-site gain, a proposed mechanism of regulatory evolution at the molecular level (see Discussion).

### Different factors have different relationships between significance and evolutionary distance

**Figure 6:**
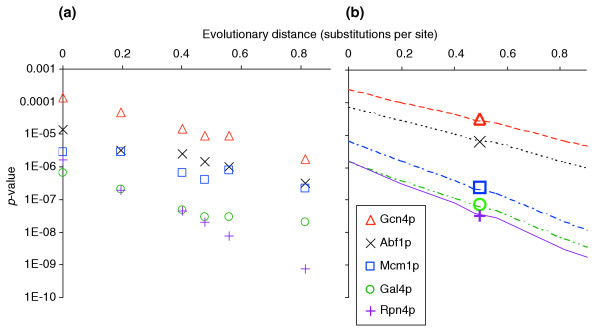
The evolutionary distance required to confidently identify conserved binding sites varies among transcription factors. **(a) **Median *p*-values for functional binding sites for various factors at different evolutionary distances. The evolutionary distance needed to obtain a desired significance varies between factors. **(b) **Predicted dependence of the *p*-values on evolutionary distance. Specificity data and functional binding sites were obtained from the SCPD.

The optimal selection of species for comparative sequence analysis remains an open question. To analyze this question for transcription factor binding sites, we examined the relationship between evolutionary distance and the MONKEY *p*-values for several *S. cerevisiae *transcription factors (Figure 6) for which sufficient characterized binding sites were available in SCPD [33]. We find that while all factors show the tendency for *p*-values to decrease with evolutionary distance, the *p*-values for each factor remain very different. For example, with alignments of four species spanning about 0.8 substitutions per site, we expect a conserved match to the Gcn4p matrix as good as the median functional binding site (Figure 6a, red triangles) approximately every million bases of aligned sequence. This in contrast to Rpn4p, for which in the same alignments we expect such a match (Figure 6a, violet crosses) only once in about 1 billion base pairs. Thus, the evolutionary distance required to achieve a desired *p*-value is different for different factors. Understanding the relationship between a frequency matrix and the behavior of its *p*-values is an area for further theoretical exploration. We note that, once again, we can predict the behavior of these *p*-values (Figure 6b), and that while our predictions agree qualitatively, there is considerable variability.

### Software

MONKEY is implemented in C++. It is available for download under the GPL and can be accessed over the web at [35].

## Discussion

By formulating the problem of identifying conserved TFBSs in a probabilistic evolutionary framework, we have both created a useful tool (MONKEY) for comparative sequence analysis capable of functioning on relatively large numbers of related species, and enabled the examination of several important questions in comparative genomics. While most previous approaches to this problem have used heuristics to define conserved and nonconserved TFBSs, with the probabilistic scores and *p*-value estimates presented here the assumptions underlying our approach can be made explicit, and where those assumptions hold we can be assured the reliability of our method. In addition, the probabilistic framework allows us to estimate the amount of evolutionary distance required to achieve a certain level of significance.

### Evolutionary models

The score based on the evolutionary model proposed by Halpern and Bruno [25] effectively discriminated the functional and nonfunctional Gal4p- and Rpn4p-binding sites in *S. cerevisiae *(Table 2). We believe the success of the HB model in predicting position-specific rates of evolution [19] and identifying conserved TFBSs reflects its encapsulation of a model of binding sites evolving under constant purifying selection. Although not every functional binding site will remain under purifying selection, as a result of either functional change or binding-site turnover (see below), a large subset of functional binding sites do remain under purifying selection, and for these, the 'HB' score performs better than the 'simple' score. It is interesting to note, however, that the simple score, which is not based on an evolutionary model and does not take into account the relationships of the species used in the comparison, still shows great improvement over one genome alone, highlighting the value of comparative sequence data even when used suboptimally.

### Effects of evolutionary distance

An important hypothesis of the comparative genomics paradigm is that as evolutionary distance increases, observing a match with a given level of conservation should become less and less likely by chance - the *p*-values for functional sites that are conserved are expected to decrease. We confirm this hypothesis for a small number of factors from *S. cerevisiae*. In addition, our probabilistic models allow us to quantify this relationship. We can directly measure the confidence that a specific site is a conserved binding site, and we can predict the evolutionary distance needed to achieve a desired level of significance.

Typical *p*-values for functional binding sites scored by matching a matrix to a single genome are on the order of 10^-4 ^to 10^-6^. Even in a relatively small genome like yeast, with roughly 12 million bases, we expect many matches at this significance level to occur by chance. Adding four closely related species that span a total evolutionary distance of approximately one substitution per site reduces these *p*-values by approximately three orders of magnitude to the range 10^-7 ^to 10^-9^. In the yeast genome we expect few, if any, matches to occur at this level of significance by chance. When we search the alignments of these species with the Rpn4p matrix with a low enough *p*-value that we expect a match at that significance to occur only once in a random 50 Mb genome, we recover nearly the entire positive set of Rpn4p-binding sites while excluding most of the negative set, highlighting the utility of MONKEY and the statistics we have developed. As a measure of the improvement over searching a single genome alone, we note that even the best possible match to the Rpn4p matrix in one genome does not meet this significance criterion.

The expected relationship between evolutionary distance and *p*-value can, in principle, be used to guide to choice of species to be sequenced for comparative analyses. However, the dependence of *p*-values on evolutionary distance is not the same for all factors (Figure 6). This suggests that our ability to annotate functional sequences by comparative methods will depend on the type of sequences that we are trying to annotate, and that there is no single evolutionary distance sweet-spot for identifying TFBSs.

### Pairwise versus multiple species comparisons

In theory, for a given reference genome it should be possible to pick a single comparison species at an evolutionary distance sufficient to identify any conserved feature of interest. Our results suggest that at distances of up to approximately 0.6 substitutions per site, pairwise alignments provide essentially the same amount of resolving power as multiple comparisons spanning the same evolutionary distance. We showed that *S. cerevisiae *and *S. kudriavzevii *span almost exactly the same evolutionary distance as *S. cerevisiae*, *S. paradoxus *and *S. mikatae*, and that that distance is well below 0.6 substitutions per site. Consistent with this, MONKEY produces nearly identical *p*-values for conserved binding sites from these two sets of species. Thus, our results suggest that from a theoretical perspective, if the goal of comparative analysis is to identify conserved binding sites for factors like the ones considered here, it is not necessary to sequence species much more closely related than this limit.

We note, however, that there are myriad practical reasons other than evolutionary resolving power (the only factor considered in our models) for sequencing multiple closely related sequences. First, there may simply be no extant species at the exact evolutionary distance desired. Second, the quality of DNA alignments is expected to be much higher for multiple closely related species than for more distant pairwise alignments - if alignment errors prevent correct assignment of orthology, conserved binding sites will not be identified. For the factors considered here, the pairwise comparison performed nearly as well as the multiple species comparison well beyond the evolutionary distances at which pairwise alignments are reliable [36], suggesting that the necessity of alignment will limit the maximum distance between species. Finally, and perhaps most important, is the assumption that our models make about constant functional constraint over evolution. To illustrate this, consider the binding sites for Gal4p used in the analysis in Figure 2a. These binding sites could not be included in Figure 3 because *S. kudriavzevii *orthologs for these genes were not available in SGD, apparently because of the degeneration of the galactose-utilization pathway in this species [37]. Sequencing multiple closely related species provides insurance against such functional changes, because they are less likely to have occurred in all the lineages.

### Conserved sites and binding-site turnover

MONKEY was very effective in identifying functional Rpn4p-binding sites from the alignment of five *Saccharomyces *species. In our search, 41 of 56 (73%) predicted sites were found near genes showing the expected expression pattern, and are therefore likely to be functional. Even at this level of stringency, however, there are highly conserved sequences that match the matrix, but do not appear to be near genes that are regulated by Rpn4p. It is very unlikely that these sites are conserved by chance. One possible explanation for this high degree of conservation is that these are functional sites, but that the expression of these genes is not accurately detected in high-throughput assays, or their function has not been accurately determined. A more likely possibility is that these sites are conserved because they perform other, unknown functions. Consistent with this hypothesis is the fact that many of these matches fall near other highly conserved sequences (Figure 4b), suggesting that they may be parts of larger conserved features.

In addition to the conserved sequences that are unlikely to represent *bona fide *binding sites, we also found examples of binding sites associated with properly regulated genes that do not seem to be conserved (Figure 5). Once again there are several possible explanations for this observation. First, these binding sites may not actually be functional and may have been included in our positive set erroneously. While this is a possible explanation for the case of the Rpn4p-binding sites shown in Figure 5 (and may be likely in the case of *REH1*, where we could identify another apparently conserved binding site in the region) we have also found nonconserved examples among the TFBSs in the SCPD database (approximately 20% of TFBSs we examined, see Additional data file 1), all of which have at least some direct experimental support.

Another potential explanation is that these binding sites are actually conserved, but were not aligned correctly. While this is difficult to rule out in general, in the few nonconserved cases for Rpn4p at least we could not find (by eye) errors in the alignments. Most interesting, of course, would be the situation where these nonconserved binding sites are not due to some error on our part, but rather represent a biological change in the functional constraints on these sequences, possibly resulting in a change in the regulation of the expression of these genes. Our results represent an upper bound on the number of TFBSs for which this has occurred. *Cis*-regulatory changes have been proposed to be an important source of genetic variation [32]. Gains and losses of functional binding sites represent an important class of these changes [38,39], and an important area for future computational and experimental analysis, particularly as the genome sequences of closely related metazoans become available. We expect MONKEY to be a useful tool in the comparative analysis of these genomes, and we have found comparable increases in the significance of functional binding sites in alignments of *Drosophila melanogster *and *D. pseudoobscura *(see Additional data file 2).

## Conclusions

We have developed a method to identify conserved TFBSs in sequence alignments from multiple related species that provides a quantitative framework for evaluating results. The method - implemented in the open-source software MONKEY - extends probabilistic models of binding specificity to multiple species with probabilistic models of evolution. We have found that a probabilistic evolutionary model [25] that assumes binding sites are under constant purifying selection performs effectively in discriminating functional binding sites. We have developed methods to assess the significance of hits, and have shown that the significance of functional matches increases while the significance of spurious matches decreases over increasing evolutionary distance. We can explicitly model the relationship between the significance of a hit and evolutionary distance, allowing the assessment of the potential of any collection of genomes for identifying conserved binding sites. Applying MONKEY to a collection of related yeast species we find that most functional binding sites are highly significantly conserved, but also find evidence for conserved sites that are not functional and *vice versa*. Our results suggest that development of methods that model the evolutionary relationships between species and the evolution of the genomic features of interest yield insight into the challenges for comparative genomics.

## Materials and methods

### Simulating pairs of sequences

To generate the empirical *p*-values shown in Figure 1, random sequences of length *w *were generated according to the average intergenic base frequencies of the *S. cerevisiae *genome. These were then evolved according to the Jukes-Cantor substitution model, to a specified evolutionary distance. Likelihood ratio scores and *p*-values were then calculated for each of the pairs of sequences using the method implemented in MONKEY. Finally, all pairs of sequences were ranked by their scores, and the rank divided by the total number of pairs was taken as the empirical *p*-value.

### Preparation of alignments for different groups of species

We aligned the upstream regions of all *S. cerevisiae *genes to their orthologs in *S. paradoxus*, *S. mikatae*, *S. bayanus *and *S. kudriavzevii *by taking the 1,000 bp upstream of each gene, identifying the corresponding region from the other species using data in the *Saccharomyces *Genome Database [40], aligning them with CLUSTAL W [41] and trimming them to remove regions corresponding to *S. cerevisiae *coding sequence. We used this strategy rather than simply aligning intergenic regions to control for differences in alignments that might arise from the use of variably sized regions.

To obtain estimates of the evolutionary distance spanned by each comparison, we ran PAML [24] on the entire set of intergenic alignments, using the HKY model [27], with constant rates across sites. We used the median PAML estimate of kappa (the transition-transversion rate ratio) of 3.8, the *S. cerevisiae *background frequencies (ACGT) = (0.3, 0.2, 0.2, 0.3) and the median of the branch lengths estimates as the 'background' evolutionary model. The trees with these branch lengths were used as input to MONKEY to calculate *p*-values. The distances in Figure 4 represent the sum of the median branch lengths in each comparison. The subsets (with evolutionary distances in parentheses) were as follows: *S. cerevisiae *and *S. paradoxus *(0.194); *S. cerevisiae *and *S. mikatae *(0.403); *S. cerevisiae*, *S. paradoxus S. mikatae *(0.477); *S. cerevisiae *and *S. bayanus *(0.559); *S. cerevisiae*, *S. paradoxus, S. mikatae *and *S. bayanus *(0.816); *S. cerevisiae*, *S. paradoxus, S. mikatae, S. bayanus *and *S. kudriavzevii *(1.090).

### Definition of Rpn4p and Gal4p matrices and positive and negative sets

**Rpn4p: **we used Rpn4p sites in proteasomal genes [42,43] to build an Rpn4p specificity matrix (using a pseudocount of 1 per base per position). To identify additional likely targets, we obtained expression data from public sources [30,31] and compared the expression patterns of all genes to the average expression pattern of proteasomal genes using the following metric:



where *θ *is the 'uncentered correlation', a commonly used distance metric for gene-expression data [44]. Our score adds a correction for the number of datapoints, *n*, that are available for each gene. All matches to the Rpn4p matrix (*S. cerevisiae *likelihood ratio score > 9) in the upstream region of a gene that matched the proteasomal expression pattern (*t *> 8) were considered to be true Rnp4p sites. The negative set consists of all sites that matched the Rpn4p matrix with a score greater than 9, and excluded sites in genes with even weak similarity to the proteasomal expression pattern (*t *> 0) or that were annotated [40] as involved in protein processing or degradation.

**Gal4p: **we used the matrix from SCPD [33] (with a pseudo count of 1 per base per position). To define a positive set we used the binding sites in SCPD and systematic studies of this Gal4p regulatory system [45,46], and used matches near additional genes that we identified in these studies with scores above the lowest score in the SCPD set. To define a negative set, we again scanned the *S. cerevisiae *genome with a cutoff equal to the lowest score in the positive set and then eliminated any binding sites near genes that showed evidence for regulation in the systematic studies.

It is important to note that our categorization of sequences as positive and negative is done independently of the comparative sequence data, thus avoiding potential circularity.

### Calculations of expected scores

Because our methods employ explicit probabilistic models for the evolution of noncoding DNA, it is possible to compute the expected scores under various assumptions. The expectation of the log likelihood ratio for examples of the motif is the 'information content' and its calculation has been addressed [47]. We can extend this to calculation to our evolutionary scores, as follows. Using the fact that all the scores treat the positions of the matrix independently, and the linearity of the expectation, we write:



where *E *[*x*] denotes the expectation of the random variable *x*, *m *denotes a frequency matrix and a corresponding evolutionary model, either {*motif*, *R*_*motif*_} or {*bg*, *R*_*bg*_}. *p*(*X*_*i*_, *Y*_*i*_|*m*, *T*) is calculated as above, and we define:



We can write a similar expression for the variance, *V*:



In order to predict the scores for the genes in our positive and negative sets, we are interested in the case were we have observed a match to the motif in one species, but the constraints on its evolution are either those of the background or the motif. We can compute the expected scores under these assumptions as follows:



where *p*(*X*_*i*_|*motif*) is the single species probability of observing the base *X*_*i *_at position *i *in the specificity matrix (*f*), and using Bayes' theorem:



This calculation can be extended to the multiple species case, by replacing the distributions *p*(*X*_*i*_, *Y*_*i*_) and *p*(*Y*_*i*_|*X*_*i*_) with *p*(*X*_*i*_, *Y*_*i*_, ..., *Z*_*i*_) and *p*(*Y*_*i*_, ..., *Z*_*i*_|*X*_*i*_) and changing the sum over *Y*_*i *_to a sum over all the other leaves in the tree except the reference, in this case, *X*_*i*_. For the functional set, we assumed the binding sites were evolving under the HB model [25], and for the nonfunctional set we assumed evolution under the HKY background model described above. To model the sequence-specificity matrices most accurately, we reduced the pseudocount (equal to the background probability of observing each base).

## Additional data files

Additional data file 1 shows the fraction of binding sites that are not conserved for several different *S. cerevisiae *transcription factors. Additional data file 2 shows the conservation p-values of predicted binding sites in high-density binding site clusters in the *Drosophila *melanogaster genome, with the binding sites grouped according to whether the cluster has regulatory activity.

## Supplementary Material

Additional data file 1 The fraction of binding sites that are not conserved for several different *S. cerevisiae *transcription factors Click here for additional data file

Additional data file 2 The conservation p-values of predicted binding sites in high-density binding site clusters in the *Drosophila *melanogaster genome, with the binding sites grouped according to whether the cluster has regulatory activity Click here for additional data file

## Figures and Tables

**Table 1:** Definition of positive and negative sets of matrix matches

Criterion	Gal4p	Rpn4p
Unique specificity	Spacer of 11 bp [50]	Atypical zinc finger [42]
Well characterized specificity	Protein-DNA co-crystal [51]	Large number of binding sites, low degeneracy [42]
Well characterized target gene set	Classic genetic system [52] and high-throughput studies [45,46]	Targets include almost all proteasomal subunits [42] stereotypical expression pattern [48]

Criteria used to define positive and negative sets to use in this study. It is important to avoid factors whose specificity overlaps with other factors, because binding sites that are not occupied by one factor may be constrained because of binding by another, and to choose factors with characterized specificity because our methods rely on the assumption that the specificity is known.

**Table 2:** Performance of different scores in recognizing functional and nonfunctional sites

Percent of binding sites assigned a lower *p*-value	Gal4p		Rpn4p	
	Positives (*n *= 10)	Negatives (*n *= 10)	Positives (*n *= 30)	Negatives (*n *= 29)
Halpern-Bruno vs simple	90%	30%	80%	25%
Halpern-Bruno vs *S. cerevisiae *alone	100%	40%	87%	34%
Simple vs *S. cerevisiae *alone	100%	30%	90%	48%

The score based on the Halpern-Bruno (HB) model assigns lower *p*-values to functional binding sites and higher *p*-values to nonfunctional binding sites than the simple score, defined as the average of the single species scores in at that position in the alignment. Both methods are far superior to *p*-values from *S. cerevisiae *alone. See text for details.
