# Supplementary material for: MONKEY: identifying conserved transcription-factor binding sites in multiple alignments using a binding site-specific evolutionary model
Source: Genome Biol. 2004 Nov 30;5(12):R98. doi: 10.1186/gb-2004-5-12-r98 (PMC545801; doi:10.1186/gb-2004-5-12-r98)
Supplement: Additional data file 1 — The fraction of binding sites that are not conserved for several different S. cerevisiae transcription factors [file gb-2004-5-12-r98-s1.pdf]

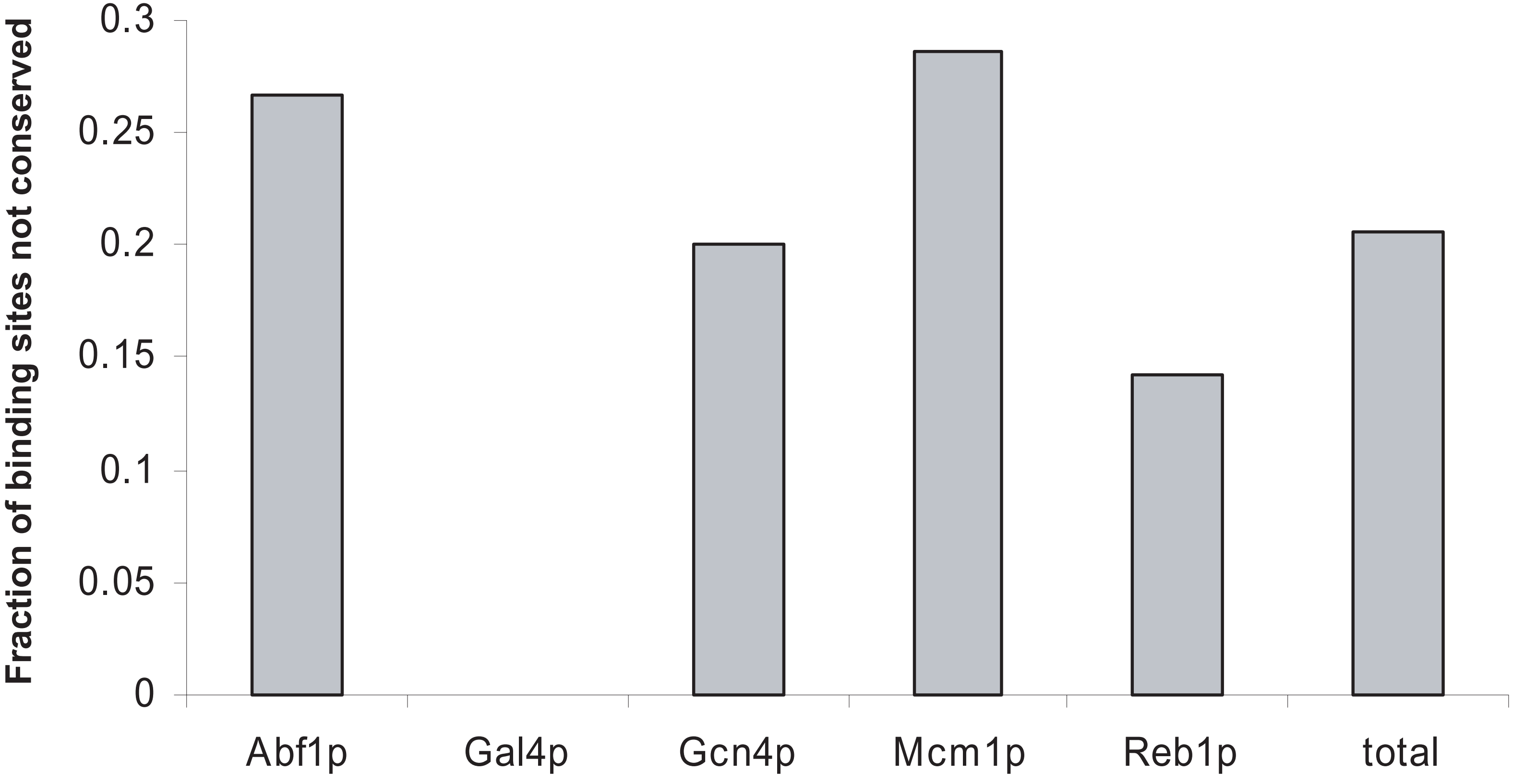

Many characterized binding sites do not seem to be evolving under constraint. We defined a binding site as not conserved if its p-value in the four way alignment of *S. cerevisiae*, *S. paradoxus*, *S. mikatae*, and *S. bayanus* was higher (less significant) than in *S. cerevisiae* alone.
