# Supplementary material for: MONKEY: identifying conserved transcription-factor binding sites in multiple alignments using a binding site-specific evolutionary model
Source: Genome Biol. 2004 Nov 30;5(12):R98. doi: 10.1186/gb-2004-5-12-r98 (PMC545801; doi:10.1186/gb-2004-5-12-r98)
Supplement: Additional data file 2 — The conservation p-values of predicted binding sites in high-density binding site clusters in the Drosophila melanogaster genome, with the binding sites grouped according to whether the cluster has regulatory activity [file gb-2004-5-12-r98-s2.pdf]

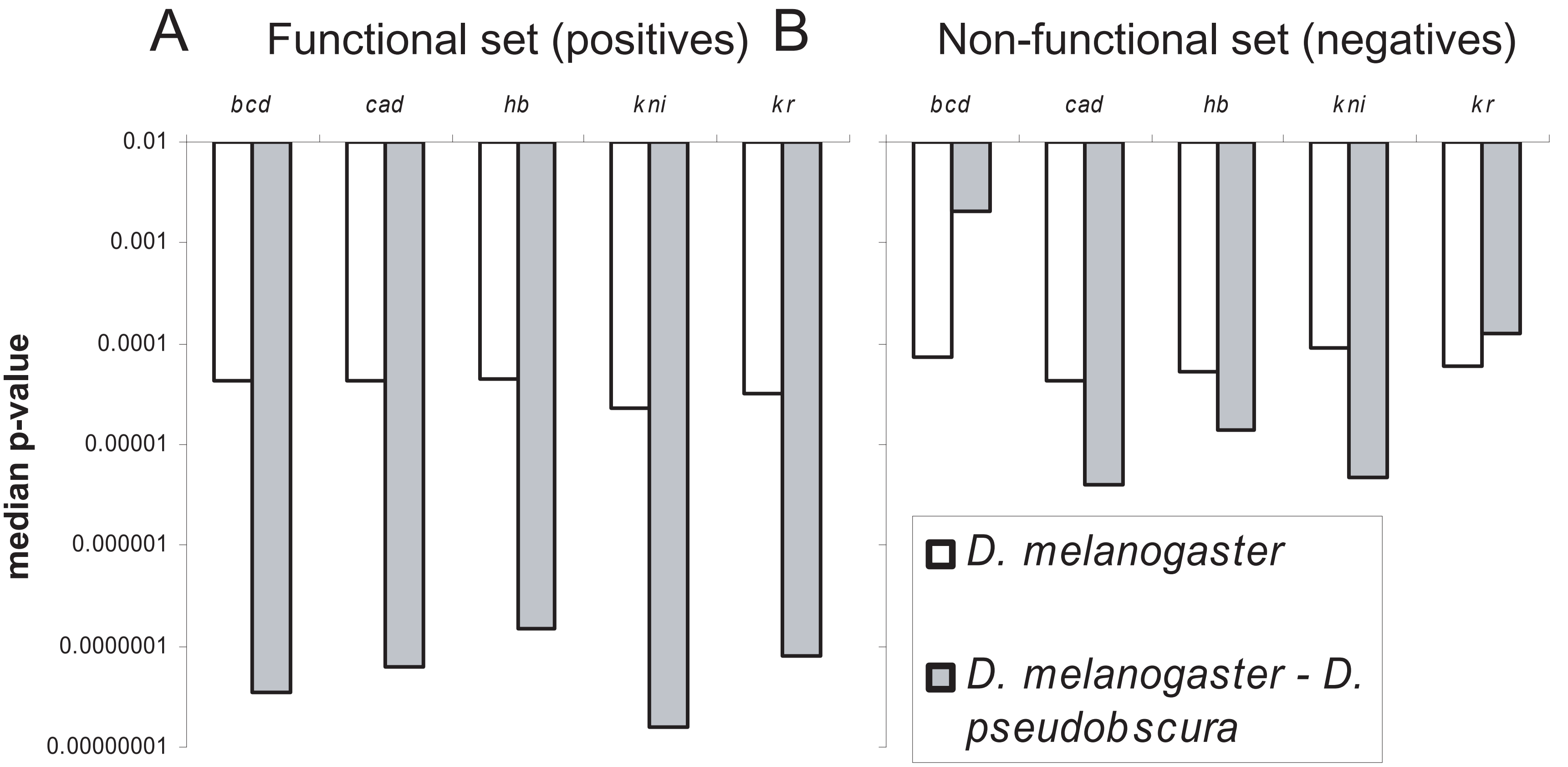

Putative binding sites (*D. melanogaster* p<0.0001) in alignments of enhancers from Berman et. al. 2004. A. The p-values of binding sites in functional enhancers decrease as expected, consistent with the hypothesis that they are under constraint. B. The p-values in the non-functional set behave somewhat more erratically, indicating that some fraction of them may be under functional constraint
